# Cases of Caries and Exfoliation of the Jaw

**Published:** 1854-07

**Authors:** W. A. Shelby


### ARTICLE VIII.

*Cases of Caries and Exfoliation of the Jaw.* By DR. W. A. SHELBY.

**Extracts from Letter.**

WARWICK, *Feb.* 19, 1854.

MR. J. W. DERR. *Dear Sir*—In compliance with your request, I hereby furnish you with an account of the circumstances attending the case of caries and exfoliation of a portion of the inferior maxilla, as far as memory serves me. Not having taken notes at the time, and a number of years having elapsed, I hope I may be pardoned for not giving the full particulars and minutiæ of the case, as might be expected.

I was summoned to see Miss E., of Elizabeth township, Lancaster county, Pa., on June 1st, 1841. Her age was 19 years; she was of a bilious temperament, and was a tall well built specimen of her sex and age. On inquiring into the history of the case, I learned that she had always enjoyed good health, up to the period of this attack; that some eight weeks previous she became afflicted with an ordinary toothache in the first inferior molar of the right side, and that in the course of a few days an extensive swelling of the corresponding cheek took place, accompanied with high inflammatory action of the general system.

Their usual family physician was called in, who treated her for a period of about seven weeks, with antiphlogistics, alteratives, rubefacients, blisters and discutients, apparently with little benefit, and eventually relinquished the case to its own course, having exhausted the catalogue of remedies.

At this juncture I was called in. She presented a pitiful appearance; her otherwise robust constitution worn down by hectic, inability to take nourishment and loss of sleep; the swelling of the cheek and part of the neck was enormous, and presented a livid mottled appearance. From her mouth flowed a constant stream of saliva and purulent matter of a very fetid character, which kept up almost constant nausea and frequent vomiting.

On examining the originally painful tooth, with the intention of removing it as the source of mischief, since it was considerably decayed, I found the soft parts around the tooth destroyed by ulceration, and a portion of the alveolar processes exposed around its roots. This is indicated in the specimen at the present day by a brownish yellow tinge.

In an attempt to shake the tooth with my finger and thumb, I found all the teeth moving simultaneously. Satisfied that a section of the bone was detached by caries and imbedded in purulent matter, I at once determined upon its removal, but not having known the nature of the case, until I arrived at the residence of my patient, I found myself unprepared to proceed that day, so I left and returned the next day, June 2d, "armed," to use the language of the late Dr. Harlan, in his remarks on Prof. Wm. Gibson's operation for osteo-sarcomatous tumor of the antrum and superior maxilla, "with horrid crooked knives, gouges, chisels, saws, and even such cruel instruments as red hot pokers," i. e., actual cautery—in short, I prepared myself to meet any emergency with regard to bones and blood vessels. I dreaded, more particularly, the inferior maxillary artery. *A priori*, it was impossible to know in what condition it might be, and a section of it must necessarily be removed with the bone.

I kept my actual cautery in a serviceable condition, and with a small scalpel made a section on each side of the teeth, from the cuspidatus to the last molar. I then grasped the denuded portion of the bone surrounding the roots of the first molar, with a pair of lower molar forceps, and by a vascillating slight traction, brought the entire diseased portion away, with the two bicuspids and two molars in their respective situations. I found much less difficulty in its removal than I had apprehended. There was considerable hæmorrhage at first, but it soon yielded to cold, astringents, such as tinct. gallæ in water, and acetat. plumbi in solution. These I ordered to be continued four or five times, daily, and after a few small spiculæ of corroded bone discharged, during the first few days, the discharge almost suddenly ceased, the loose flabby gums contracted and approximated, and new granulations of flesh sprouted from the bottom, closing the wound in a remarkably short time. Every other distressing symptom vanished, her stomach regained its tone and tranquillity, and her general system was soon renovated.

During all this improvement of symptoms, I felt deep regret and anxiety for her future appearance, apprehending an awful deformity from collapse of the cheek in accommodating itself to the vacuum thus formed inside. The cheek, as I have already stated, was very much enlarged and indurated, and was very tardy in resolving itself. Even after the removal of the bone and the subsidence of every other unpleasant symptom, it required several months for its gradual resolution, and about the time I was watching for the deformity to take place, I was most happily disappointed by finding that nature had most mysteriously been busy during this time in building a bony bridge across the lower part of the chasm, thereby effectually preventing collapse of the cheek and saving the young lady from a very unpleasant disfiguration, at the same time giving strength, permanency and symmetry to the entire inferior maxilla and ensuring the due performance of its office in mastication.

What became of the duct of Steno and other salivary ducts, I cannot precisely tell, but my impression is, that they must in a great measure have become obliterated and destroyed in common with the inferior maxillary artery and nerve—through inflammation, ulceration and subsequent cicatrization, &c.—but one thing is certain, she did not suffer any subsequent inconvenience from the destruction of parts, with the exception of having her upper grinders rendered useless by the loss of their antagonists below. Her digestive powers were good and her well nourished appearance and rosy cheeks betokened a sound state of her constitution when I saw her last in the spring of 1845, a short time previous to her removal with her father's family to Fort Wayne, in the state of Indiana, where, I am lately informed by her relations, she is still living in the enjoyment of good health.

Mr. J. E., a first cousin to the young lady whose case I have just stated, a blacksmith by trade, aged about 32 years, called upon me early one October morning, about eight years ago. His appearance was ghastly pale and betokened great suffering; he informed me that he had suffered tooth and jaw-ache, extending into the temporal region and ear, for three successive days and nights unremittingly, that he could neither sleep, eat nor rest any where. The right inferior dens sapientiæ was very far gone by caries and was very sore; the gums slightly inflamed and swollen, so was the external cheek and the parotid and submaxillary glands of the side.

I lanced the gums and removed the tooth with the forceps, but when I examined it, found one fang behind. This was owing to one half of the crown of the tooth having been destroyed by caries down to the separation of the roots--the portion which I removed came out with ease, and in a few minutes my patient was free from pain, became very cheerful and took a hearty breakfast with me, the first meal in three days. As he was now relieved, he concluded to go to the city of Lancaster that day upon a business errand, eight miles distant. The day was rather cool and damp, occasionally raining. Whether he actually got wet I do not recollect, but at all events, it was an imprudent exposure under the circumstances, and on the following night the pains recurred—he summoned me to call on him and bring something to destroy the nerve in the remaining fang. I attempted its removal but owing to its depth and the swelling and sensibility of the soft parts, I soon gave up the idea and resorted to blood-letting, blistering, purgatives, diaphoretics, alteratives and anodynes, all in their turn. The pain and swelling gradually increased instead of diminishing, and the swelling eventually extended up to the vertex and towards the occiput. Only temporary relief was afforded occasionally by the administration of morphia and camphor. The case proved so obstinate and the suffering so great that one physician after the other was called into consultation until we numbered four, amongst whom I would mention the names of my friends, Dr. John L. Atlee, of Lancaster city, and Dr. Isaac Winters, of Hinkletown, Lancaster co., two experienced and accomplished physicians as are to be found any where—but all our united efforts were in vain; our patient soon became phrenzied from meningeal congestion, and before we could give vent to any deep-seated collections of matter which we suspected under the temporal fascia, he died, after suffering about three weeks. No autopsy was allowed.

